# Evaluating and Improving the Quality of the Follow-Up Checklist at Dongola Specialised Hospital: A Clinical Audit

**DOI:** 10.7759/cureus.75266

**Published:** 2024-12-07

**Authors:** Abubakr Muhammed, Mohaned Altijani Abdalgadir Hamdnaalla, Fakher Aldeen Raft Fakher Aldeen Noman, Mustafa Mohamed, Mohammed Ali Mohammed Ali, Mohammed Maher Abdallah Awadelkareem, Moazer Ibrahim Hamid Mohammed, Ibrahim Awad, Raeda Eshag Abdualhi Ali, Noureldin Mustafa Noureldin Mustafa, Maithaa Elwlid Abdelhay Mahmoud, Reham Abdalla Bakri Mohamed, Mohamed Abdalla Elawad Wedatalla, Amal Ibrahim Abdalla Ibrahim, Mayada Elwalid Abdelhay Mahmoud, Sara Mahmoud Ali Gagar, Ahmed Elfatih Fagier Mohamed, Faris Jamalaldeen Mohammed Hamed

**Affiliations:** 1 Surgery, University of Gezira, Wad Madani, SDN; 2 Internal Medicine, Dongola Specialised Hospital, Dongola, SDN; 3 Internal Medicine, Al-Neelain University, Khartoum, SDN; 4 Microbiology, Al-Neelain University Faculty of Medicine, Khartoum, SDN; 5 General Surgery, Al-Neelain University, Khartoum, SDN

**Keywords:** audit cycle, clinical notes, follow-up, internal medicine, quality improvement

## Abstract

Background: Clinical notes are essential for patient care, guiding treatment decisions, and supporting research. This study explores how structured documentation impacts the quality of clinical notes in resource-limited settings like Sudan.

Materials and methods: This retrospective-prospective clinical audit was conducted in the Internal Medicine Department at Dongola Specialised Hospital. A checklist, developed using the National Institute for Health and Care Excellence (NICE) guidelines, assessed 10 key documentation areas, including patient history, vital signs, and management plans. Data from 50 randomly selected clinical notes were analyzed for each of two audit cycles: July 2024 (retrospective) and September 2024 (prospective, following the implementation of a structured template and staff training). Statistical analysis was employed to evaluate compliance improvements across cycles.

Results: Compliance improved significantly from 31.1% in the first cycle to 84.9% in the second. Specific improvements included chief complaint documentation (22% to 92%), current medications (18% to 86%), and lab results (12% to 86%). These findings highlight the positive impact of structured templates and targeted staff training on documentation quality.

Limitations: The study's findings are limited by its small sample size, which may not fully represent the broader patient population. Future research should address this limitation to validate and generalize the results.

Conclusion: Implementing a standardized template and training improved the quality of medical documentation at Dongola Specialised Hospital.

## Introduction

Clinical notes function as a documentation of the care administered to a patient, rendering them essential. This record can monitor decisions for future therapy, alterations in a patient's state, and the progression of a disease or ailment. Clinical notes are also crucial for research and teaching since they provide a comprehensive record of a patient's condition and course of therapy. Clinical notes can significantly affect the standard of care patients receive and are an essential component of medical records. This is why it is crucial to select clinical notes that satisfy doctors' and patients' demands. Maintaining precise and current clinical notes enables healthcare workers to deliver optimal treatment to their patients [1].

To improve documentation efficiency and reduce the amount of time doctors must spend on documentation, several technologies and methods have been created in recent years. Content importation technology (CIT) is the term used to describe these methods. Examples of CIT include templates, macros, automatic data input from other sections of the electronic health record (EHR), and copy-and-paste functions (CPF). Faster recordkeeping during patient visits appears to be one of the many advantages of these systems. However, according to Weis and Levy, there are several hazards associated with using CIT. A reader may become distracted from important, crucial facts and data if information from other sections of the record is incorrectly included or if the notes are overly lengthy and bloated [2]. However, these hazards should be manageable when utilized appropriately.

The efficiency of documentation must be improved, but accuracy of documentation is also required. Cohen et al. emphasized the need for more standardization of documentation, stating that physician variance in EHR documentation impedes the safe and effective use of EHRs [3]. According to certain research, however, standardized and organized documentation, henceforth referred to as structured documentation, may hinder note expressivity. In exploring this conflict between organized and flexible narrative documentation, Rosenbloom et al. suggested that healthcare personnel should be free to document patient care following workflow and note content requirements [4]. This suggests that when it is desirable to reuse data, organized documentation is the better option. However, narrative documentation might be employed when information reuse is unnecessary.

According to research [5], structured documentation can reduce documentation time and increase provider efficiency. Despite the increasing significance of structured documentation for data reuse, the main objective of clinical note documentation is to enable high-quality patient care [6]. Unfortunately, little is known regarding how the quality of clinical notes is affected when structured and standardized clinical note documentation replaces largely unstructured, free-text clinical note documentation.

Despite the advantages of CIT, it requires good resources for its implementation. Many developing countries, such as Sudan, face significant challenges in providing this technology across all hospitals. As a result, the healthcare system in these countries often resorts to traditional methods, which are still used in a large number of hospitals in Sudan. Therefore, the main goal was to determine how the quality of clinical notes was affected by more standardized and structured recordings.

## Materials and methods

This retrospective-prospective study was undertaken in the Internal Medicine Department of Dongola Specialised Hospital, Northern Sudan State, a large urban hospital with over 90 beds. Dongola Specialised Hospital's Institutional Review Board obtained ethical permission for this study. Our study aimed to assess the quality of follow-up clinical notes using a standardized structured template in light of the National Institute for Health and Care Excellence (NICE) requirements, identify documentation gaps, and implement organized interventions to improve the proforma format. Table 1 highlights the parameters used in this clinical audit.

**Table 1:** The parameters of the follow-up checklist as suggested by the National Institute for Health and Care Excellence (NICE) guidelines Reference: [1]

Parameters Checklist
Date and time
Chief complaint (CC)
History of present illness (HPI)
Past medical history (PMH)
Current medications
Vital signs
Physical examination
Laboratory results
Diagnosis
New plan of management

Aims and objectives

This clinical audit aims to evaluate and improve the quality of follow-up clinical notes in the Internal Medicine Department, identify problems, and provide practical suggestions based on best practices. By addressing documentation gaps, this study aims to improve clinical practices and patient safety.

Study design and population

The audit consisted of two cycles: a retrospective review of clinical notes in July 2024 and a prospective review of notes in September 2024, following the implementation of interventions. A total of 100 follow-up clinical notes were analyzed, with 50 notes evaluated in each cycle.

Sampling and sample size

A simple random sampling technique was used to ensure that the selected follow-up clinical notes were representative of cases managed by the department. The sample size of 50 notes per cycle was based on feasibility and the department's caseload during the study period. Although this sample size was practical, no formal sample size calculation was conducted, which is a potential limitation of the study.

Inclusion and exclusion criteria

The inclusion criteria included all follow-up clinical notes from patients managed in the Internal Medicine Department during the study periods. Notes from non-medical specialties, emergency cases, and incomplete or illegible records were excluded to maintain consistency and accuracy in data analysis.

Added cycles

First Cycle

The first cycle was conducted retrospectively in July 2024. A total of 50 follow-up clinical notes were reviewed using a checklist developed according to the NICE guidelines. The checklist evaluated 10 parameters, including documentation of date and time, chief complaint, history of present illness (HPI), vital signs, and management plans. This cycle aimed to establish baseline adherence to documentation standards, highlighting areas requiring improvement.

Intervention Details

Based on the findings from the first cycle, interventions were implemented to address identified deficiencies. These included the introduction of a revised documentation template and a structured training program for staff. The updated template emphasized critical documentation elements, such as the history of the present illness, medication details, and laboratory results. Training sessions, each lasting one hour, were conducted over two weeks and included practical examples, case-based discussions, and feedback on common documentation errors.

Second Cycle

The second cycle was conducted prospectively in September 2024, following the implementation of interventions. Another 50 follow-up clinical notes were reviewed using the same checklist to ensure consistency in evaluation. This cycle aimed to measure the impact of the interventions on compliance with documentation standards.

Checklist development and data collection

The checklist used for this audit was developed following the NICE guidelines and consisted of 10 parameters: date and time, chief complaint, HPI, past medical history, current medications, vital signs, physical examination, lab results, provisional diagnosis, and management plan. Data collection was carried out by trained medical personnel. To ensure reliability, two independent reviewers assessed 20 randomly selected notes from each cycle. Inter-rater reliability was evaluated using Cohen's kappa, achieving a score of 0.85, indicating strong agreement.

Statistical analysis

Data were analyzed using Microsoft Excel 2016 (Microsoft Corporation, Redmond, Washington, USA). Descriptive statistics, including frequencies and percentages, were used to summarize data. Paired t-tests were employed to compare compliance rates between the two audit cycles for each parameter, with a significance level set at p < 0.05.

Ethical considerations

Ethical approval for this audit was obtained from the Institutional Review Board of Dongola Specialised Hospital under IRB number (DO2024/014).

## Results

This study assessed the quality of medical documentation at Dongola Specialised Hospital by comparing two audit cycles. The results reveal significant improvements across various documentation criteria, reflecting the positive impact of the implemented interventions.

The documentation of the new chief complaint demonstrated a dramatic increase from 22% in the first cycle to 92% in the second cycle, resulting in a remarkable 70% improvement. This substantial enhancement indicates that healthcare providers are now more effectively capturing the primary concerns of patients upon admission, which is critical for guiding clinical decision-making. Similarly, compliance in documenting the history of the present illness improved from 22% to 68%, reflecting a 46% increase. This improvement suggests a better understanding and communication of patients' current health status, allowing for more tailored management strategies.

Additionally, the documentation of past medical history rose from 32% to 76%, a 44% improvement. This increase is essential for identifying pre-existing conditions that may affect treatment plans and overall patient outcomes. The recording of current medications also saw a significant rise from 18% to 86%, marking a 68% increase. Accurate medication documentation is vital for preventing drug interactions and ensuring safe prescribing practices, highlighting a commitment to patient safety.

Although documentation of vital signs was initially high at 70%, it increased to 96%, resulting in a 24% improvement. This ongoing emphasis on vital signs is crucial for monitoring patient stability and identifying potential complications early. The documentation of physical examinations also showed substantial improvement, rising from 42% to 90%, a 48% increase. Comprehensive physical assessments are essential for accurate diagnosis and treatment planning.

Notably, compliance with documenting lab results experienced the most significant improvement, increasing from 12% to 86%, representing a remarkable 74% rise. This finding highlights the hospital's commitment to integrating laboratory data into clinical assessments, thereby enhancing overall patient management. The documentation of new provisional diagnoses also saw a significant increase, rising from 10% to 82%, which indicates that clinicians are more consistently formulating and documenting provisional diagnoses. This practice is critical for guiding treatment and follow-up care.

Finally, the documentation of new plans of management improved from 50% to 88%, reflecting a 38% increase. This improvement ensures that care strategies are clearly communicated and implemented, facilitating better patient outcomes. Overall, the findings from this audit cycle demonstrate substantial enhancements in the quality of medical documentation at Dongola Specialised Hospital. These improvements underscore the effectiveness of the revised protocols and training initiatives. Future audits should continue to monitor these areas to maintain and further enhance compliance, particularly in documenting current medication, new provisional diagnoses, and lab results, which had the lowest initial compliance rates. Table 2 illustrates the percentages of documentation between the cycles and the corresponding percentage of improvement.

**Table 2:** Documentation in the first and second cycles, with the percentage of improvement

Criteria	First cycle	Second cycle	Improvement
Criteria	First cycle	Second cycle	Improvement
New chief complaint	11 (22%)	46 (92%)	70%
History of present illness	11 (22%)	34 (68%)	46%
Past medical history	16 (32%)	38 (76%)	44%
Current medication	9 (18%)	43 (86%)	68%
Vital signs	35 (70%)	48 (96%)	24%
Physical examination	21 (42%)	45 (90%)	48%
Lab results	6 (12%)	43 (86%)	74%
New provisional diagnosis	5 (10%)	41 (82%)	72%
New plan of management	25 (50%)	44 (88%)	38%

## Discussion

The study presents a notable improvement in the quality of medical documentation at Dongola Specialised Hospital, as evidenced by comparing two audit cycles. This has shown improvement from 31.1% compliance in the first cycle to 84.9% in the second cycle, demonstrating significant improvements in the quality of medical documentation and highlighting the positive impact of the interventions implemented between the two audit cycles. This change is substantial not only in terms of percentage improvements across various documentation criteria but also in its broader implications for clinical practice, patient care, and health system efficiency.

This is comparable to another study, which revealed that the average quality score for unstructured notes was 64.35%. Structured and standardized documentation increased the note quality score by 77.2%. Both studies highlight the value of structured documentation. The disparity between the two findings in the first cycle reveals the deficiencies reflected by our research, highlighting the importance of using structured documentation in our practices [7]. In addition, another study demonstrated a 56% improvement in compliance with NMBI (The Nursing and Midwifery Board of Ireland) documentation standards after implementing the education program and SOAP (subjective, objective, assessment, and plan) Notes Framework [8].

A notable improvement in documentation was observed in the chief complaint, which increased from 22% to 92%, marking a 70% improvement. This is a crucial outcome, as the chief complaint is the starting point for formulating a patient's clinical management plan. Accurate documentation of the chief complaint is essential for ensuring that healthcare providers can focus on the patient's primary concerns, which is the foundation for clinical decision-making and subsequent diagnostic and treatment decisions. This dramatic increase suggests that interventions, likely focused on clinical training or workflow changes, have directly impacted clinicians' ability to capture the most pertinent information about a patient's condition early in their care. A study by Shirazi and Masood indicated that 91.3% addressed the chief complaint when utilizing structured templates for the follow-up, which is consistent with the value of using structured templates [9].

Similarly, the HPI and past medical history substantially improved (46% and 44%, respectively). These sections of the medical record provide a critical context for understanding the patient's current health status, including the evolution of their illness and any pre-existing conditions that may complicate or influence treatment. The improvement in documenting the HPI reflects better clinician-patient communication and a deeper understanding of the clinical context, which directly contributes to more individualized treatment strategies. This was confirmed in a previous study conducted by Mazer et al. [10].

In the first cycle, the documentation of current medication data was notably weak, with a completion rate of only 18%. This low percentage highlights significant gaps in accurately filling out patient follow-up information. However, by the second cycle, this figure improved dramatically to 86%, underscoring the effectiveness of utilizing a structured template for documentation. This improvement is critical, as medication errors continue to be a leading cause of morbidity and mortality in healthcare settings, and proper documentation is key to minimizing these risks [11].

The documentation of vital signs improved by 24%, from 70% to 96%. Vital signs, such as heart rate, blood pressure, and respiratory rate, are essential for monitoring a patient's stability and identifying early signs of deterioration. The marked improvement suggests that the interventions may have emphasized the importance of timely and accurate monitoring, which is crucial for identifying potential complications in hospitalized patients. Similarly, the physical examination documentation improved by 48%, from 42% to 90%. Comprehensive physical exams provide essential information that, in conjunction with other clinical data, informs diagnoses and treatment plans. The increase suggests that clinical staff consistently perform and document thorough physical assessments, likely reflecting better adherence to clinical guidelines and improved training in comprehensive patient evaluations.

The most striking improvement was observed in the documentation of lab results, which surged from 12% to 86%, indicating a stronger integration of laboratory data into clinical assessments and decision-making. Similarly, the documentation of new provisional diagnoses and management plans saw marked increases, from 10% to 82% and 50% to 88%, respectively. These improvements suggest clinicians are more consistently formulating and documenting provisional diagnoses and care strategies, which are critical for guiding patient care and follow-up.

Overall, the findings indicate that the interventions, such as revised protocols and training initiatives, have been effective in improving the quality of medical documentation at the hospital. However, areas with low compliance, such as the HPI, past medical history, and provisional diagnoses, should continue to be prioritized in future audits to ensure sustained improvement in clinical documentation practices.

The documentation of vital signs improved by 24%, increasing from 70% to 96%. Vital signs, including heart rate, blood pressure, and respiratory rate, are crucial for monitoring a patient's stability and detecting early signs of deterioration. Previous research has consistently highlighted a significant gap in the accurate recording of vital signs, particularly the respiratory rate [12], which is critical in identifying early signs of clinical deterioration [13]. Despite its importance as a key indicator for detecting serious complications such as respiratory failure, sepsis, or cardiac arrest, the respiratory rate is often underreported or neglected in clinical practice. This oversight can delay identifying at-risk patients and hinder timely interventions, underscoring the need for improved monitoring and documentation practices to enhance patient safety and outcomes.

Impact on clinical practice

The interventions introduced in this study have the potential to lift clinical practice to new standards of accuracy and efficiency. By standardizing the documentation process, we ensure that essential clinical data are captured more consistently and comprehensively, reducing the risk of errors and omissions. Moreover, the educational component of the intervention ensures that clinicians are not only adhering to new templates but also understanding the importance of quality documentation in patient care. This shift towards a more structured approach enhances both the quality of documentation and the overall effectiveness of clinical practice, contributing to better patient outcomes and improved healthcare delivery.

Maintaining these results

To sustain the improvements achieved in this study, it is essential to implement a system of continuous monitoring and reinforcement. Regular follow-up audits and refresher training sessions should be planned to ensure that the improvements are maintained over time. Ongoing support is critical; without it, clinicians may revert to old practices, as observed in other healthcare settings. Therefore, it is recommended that Dongola Specialised Hospital implement regular audits and training updates to ensure the continued success of the documentation improvements.

Implementing similar interventions in other hospitals, particularly those in resource-limited settings, is both feasible and beneficial. The structured documentation templates used in this study are simple, cost-effective, and adaptable to different clinical environments. Despite not measuring the direct impact on patient outcomes, it is clear that improved documentation will enhance patient care by ensuring accurate and consistent recording of clinical data. The cost-effectiveness of this intervention is a key strength; it involved a simple modification, printing a new file template, that is inexpensive and easy to implement. Such a low-cost intervention has the potential to prevent catastrophic failures in patient care due to missed or incomplete documentation.

Limitations

This study has several limitations, including a small sample size without formal calculation, limiting generalizability. It did not analyze implementation challenges such as staff workload or technical issues, nor did it address cost implications or the sustainability of improvements over time. Additionally, staff feedback and acceptance of the new documentation system were not collected, which could have provided valuable insights for refinement. Importantly, the study focused on compliance with documentation standards but did not assess whether improved documentation translated into better patient outcomes, leaving it unclear if the changes enhanced patient care or simply addressed compliance.

## Conclusions

This study demonstrates significant improvements in the quality of medical documentation at Dongola Specialised Hospital, evidenced by a substantial increase in compliance across multiple documentation parameters following the introduction of a structured template and staff training. These findings highlight the value of standardized documentation and targeted training in enhancing clinical note quality.

To build on these results, hospitals in similar settings are encouraged to adopt structured templates aligned with established guidelines, coupled with regular staff training to ensure sustained improvements. Future audits should be planned to monitor the long-term effectiveness and sustainability of these interventions. Additionally, hospitals should establish clear implementation timelines for adopting these changes to facilitate smooth integration into clinical practice. Further research should explore the impact of improved documentation on patient outcomes to assess whether these changes lead to tangible benefits beyond compliance.
